# Development of a Piezoelectric-Driven XYθ_z_ Nano-Positioning Stage with High Load-Bearing Capacity Enabled by Over-Constrained Guiding Configuration

**DOI:** 10.3390/mi16050548

**Published:** 2025-04-30

**Authors:** Bin Liu, Lingchen Meng, Shuaishuai Lu, Fei Wang, Pengbo Liu, Peng Yan

**Affiliations:** 1 Shandong Key Laboratory of CNC Machine Tool Functional Components, School of Mechanical Engineering, Qilu University of Technology (Shandong Academy of Sciences), Jinan 250353, China; 18554360531@163.com (B.L.); wf@qlu.edu.cn (F.W.); 2 Key Laboratory of High-Efficiency and Clean Mechanical Manufacture, Ministry of Education, School of Mechanical Engineering, Shandong University, Jinan 250061, China; mlc_sdu@foxmail.com (L.M.); yanpeng@sdu.edu.cn (P.Y.); 3 Shandong Institute of Mechanical Design and Research, Jinan 250031, China; luss_me@qlu.edu.cn

**Keywords:** hybrid amplification mechanism, over-constrained, high load bearing, out-of-plane deformation, nanopositioning stage

## Abstract

A novel over-constrained XYθ_z_ nano-positioning stage with a high load-bearing capacity is proposed. This serially connected displacement stage adopts an embedded structural design that integrates a translation stage with a rotation stage in series. The Z-axis amplification mechanism employs out-of-plane actuation, realising a compact solution for three-axis independent motion. The hybrid amplification mechanism designed in the translation stage ensures enhanced output displacement and structural stiffness. The hybrid-parallel amplification mechanism comprises a lever-type displacement amplifier and a Scott–Russell displacement amplifier connected in series, which is then connected in parallel with a bridge-type displacement amplifier. An over-constrained mechanism is introduced to impose redundant constraints along the Z-axis, effectively suppressing parasitic displacement in the Z-direction while enhancing resistance to out-of-plane deformation. A quasi-static model of the XYθ_z_ motion stage was established to comprehensively characterise the deformation behaviour of the stage, which was verified by finite element simulations and experiments on the prototype. The experimental results indicate that the XYθ_z_ stage achieves a large motion range (up to 152.22 μm × 151.3 μm × 2.885 mrad) while maintaining excellent anti-deformation capability 200 nm at 4 kg loading.

## 1. Introduction

The flexure-based three-degree-of-freedom (3-DoF) XYθ_z_ nano-positioning stage has been widely adopted in various applications including nanoimprint lithography, scanning probe microscopy systems, and high-precision optical image stabilisation systems [1,2].

Based on this research context (as shown in Table 1), modern precision positioning systems impose stringent multi-dimensional accuracy requirements on motion stages: they must guarantee nanometre-level in-plane positioning accuracy while rigorously controlling out-of-plane deformation under loaded conditions. Particularly in nanoimprint lithography processes, when performing wafer alignment and imprinting operations across a hundred-micrometre range, the platform must simultaneously achieve nanometre-scale in-plane alignment precision while maintaining out-of-plane deformation within nanometre tolerances under imprinting forces–a dual requirement that critically determines pattern transfer fidelity [3,4]. Consequently, the development of XYθ_z_ positioning stages featuring large travel ranges, high load capacity, and superior out-of-plane deformation resistance has emerged as a pivotal technological breakthrough for meeting advanced precision manufacturing demands. Through innovative mechanism design, this study has substantially enhanced the platform’s full-range motion stability under imprint loading conditions while dramatically improving its out-of-plane deformation resistance.

Its three-degree-of-freedom (3-DoF) motion capability enables the coordinated compensation of planar position and orientation. Various XYθ stages based on amplification mechanisms, such as lever or bridge designs, have been developed, each exhibiting distinct advantages in motion stroke and decoupling accuracy. Cai et al. [8] developed an XYθ fretting stage using T-shaped flexible hinges, achieving a working range of 6.9 µm × 8.5 µm × 289 µm. Wang and Zhang [9] proposed an XYθ positioning micro-motion stage with a working range of 37.3 µm × 44.2 µm × 2200 µrad. Park et al. [10] introduced an XYθ micro-motion stage driven by a piezoelectric actuator, featuring an amplification ratio of approximately 3.0 and a first resonance frequency of 108 Hz. Lee et al. [11] optimised the design of an XYθ mask alignment table using three parallel bridge mechanisms. Additionally, some XYθ micro-motion stages employ direct-drive units for actuation. Al-Jodah et al. [12] utilised a voice coil motor to drive a prism–prism–rotation joint structure, enabling a wide motion range in XYθ_z_. Wang et al. [13] developed a novel rigid–flexible coupling 3-DoF nano-positioning stage with a high positioning accuracy and load-bearing capacity.

Although existing 3-DoF nano-positioning stages deliver large motion ranges and excellent decoupling performance, they still exhibit inherent movement constraints. The out-of-plane deformation resistance of the stage critically determines the contact uniformity between the template and wafer; any local unevenness may induce pressure variations that ultimately lead to pattern defects [14,15,16,17]. This necessitates nanoscale-precision control of out-of-plane deformation under load. The bearing system should exhibit high stiffness and precise motion control, particularly in planar 3-DoF XYθ_z_ displacement stages, to withstand external forces and vibration interference, ensuring process consistency and reliability [3,18].

This study proposes a novel 3-DoF compatible mechanism for the XYθ_z_ nano-positioning stage to enable in-plane scanning. To achieve an extended motion stroke, a hybrid amplification mechanism with parallel two-stage amplification is introduced. The over-constrained principle is employed to mitigate the impact of axial bearing stiffness on motion stiffness during bearing movement, thereby enhancing load-bearing capacity and maintaining stage flatness. The guide mechanism is integrated with the drive output to minimise parasitic motion while embedding the θ_z_ stage within the XY stage ensures a compact structure. Based on the force–displacement relationship of the stage’s drive mechanism, the kinematic model of each axis is established. The stiffness of the guide mechanism is characterised using a compliance matrix, and the output stiffness of the stage’s amplification mechanism is derived.

This study proposes an innovative three-degree-of-freedom (XYθ_z_) compliant nano-positioning stage design, with its core innovations summarised as follows:

- A hybrid serial-parallel two-stage amplification mechanism is employed, combining optimised lever amplification and bridge-type amplification designs to significantly enhance the XY stage’s motion range;
- Innovative application of the over-constraint principle effectively suppresses the negative impact of Z-direction bearing stiffness on the system’s motion stiffness. While maintaining out-of-plane deformation performance, the design substantially improves load-bearing capacity.

The remainder of this paper is structured as follows: Section 2 presents the mechanical design of the XYθ_z_ nano-positioning stage. Section 3 and Section 4 provide the corresponding analysis and finite element analysis (FEA), respectively. Section 5 introduces and discusses the experimental results, offering a comprehensive evaluation of performance. Finally, Section 6 summarises the main conclusions.

## 2. Mechanical Design

**Figure 1:**
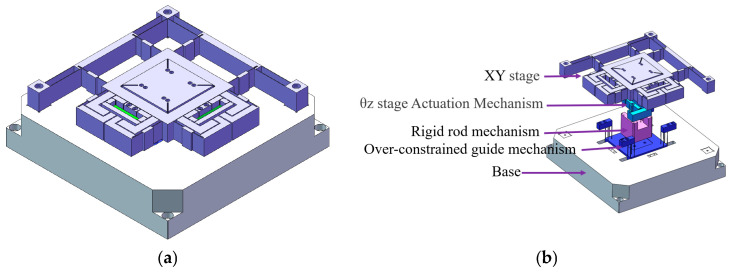
XYθ_z_ nano-positioning stage. (**a**) Isometric view. (**b**) Exploded diagram and component breakdown of the XYθ_z_ nano-positioning stage.

Figure 1 illustrates the proposed XYθ_z_ nano-positioning stage and comprises an XY translation unit, θ_z_ rotation drive unit, and z-direction over-constrained mechanism.

**Figure 2:**
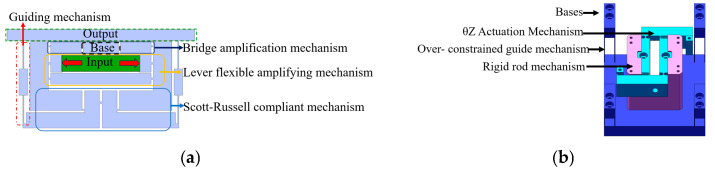
Drive unit of the XYθ_z_ nano-positioning stage. (**a**) XY hybrid amplification mechanism. (**b**) θ_z_ amplification mechanism and over-constrained mechanism.

**Figure 3:**
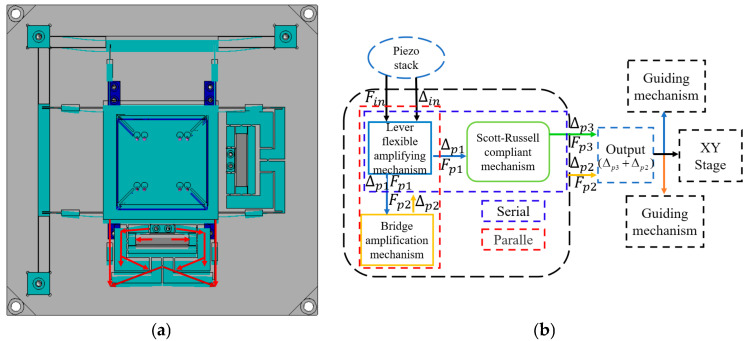
X/Y directional movement in XYθ_z_ nano-positioning stage. (**a**) Principle of X/Y translational motion. (**b**) Transmission of X/Y translational motion.

The hybrid amplification mechanism of the translation stage integrates a lever, a Scott–Russell (SR) displacement amplifier, and half-bridge mechanisms. The half-bridge mechanism is secured to the base with bolts as a fixed end, while the guide mechanism, incorporating a straight-beam leaf spring and a parallel double-parallelogram flexible mechanism, is symmetrically arranged to minimise parasitic movement in undesirable directions (Figure 2a). These ceramics apply force and displacement to the input terminal of the primary lever amplification mechanism. The amplified displacement at the output terminal is then transmitted to the parallel secondary amplification half-bridge mechanism. Subsequently, the displacement from both the lever and bridge mechanisms is directed into the series secondary amplification mechanism (SR amplification mechanism). The output displacement of the parallel secondary amplification mechanism (lever + bridge mechanism) acts on the entire drive system, enabling movement of the XY stage. Concurrently, the output displacement of the series secondary amplification mechanism (lever + SR mechanism) drives the XY stage actuator. The total output displacement of the hybrid secondary amplification mechanism is the sum of the displacements from the parallel and series secondary amplification mechanisms, enabling the nano-positioning stage to achieve an extended translation stroke shown in Figure 3.

The amplification mechanism of the rotating stage contains two parallel lever displacement amplification mechanisms. Its guide mechanism is embedded within the actuator of the translation stage, ensuring that rotational and translational movements remain coplanar, thereby reducing non-planar parasitic displacement of the stage. The Z-direction over-constrained mechanism consists of a rigid support connector and Z-direction guiding mechanism, with the rotary stage integrated within the rigid support body connecting the translation stage and the guiding mechanism. The guiding mechanism uses 16 slender straight beams symmetrically distributed at four corners, achieving complete motion decoupling in the XY directions while maintaining the axial load-bearing capacity. An over-constrained design requires precise balancing between degrees of freedom and constraints. The core principle involves first defining the desired degrees of freedom before applying constraints to non-motion directions. However, increased motion range may lead to amplified parasitic errors and enhanced axial coupling effects, and excessive symmetric constraints may limit motion flexibility.

**Figure 4:**
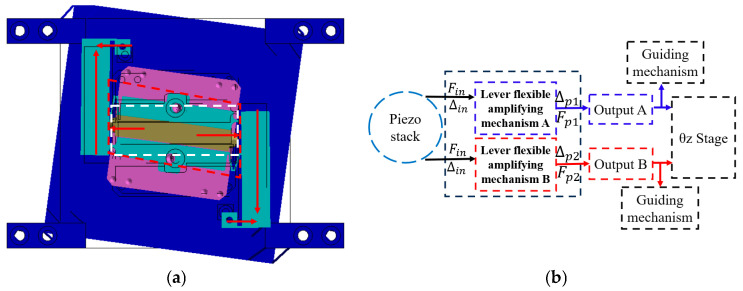
θ_z_ directional movement in XYθz nano-positioning stage. (**a**) Principle of θ_z_ rotational movement. (**b**) Transmission of the θ_z_ rotational movement.

The XYθ_z_ stage designed in this study has X/Y translation and θ_z_ rotation degrees of freedom. It enhances Z-direction stiffness using 16 symmetrically arranged slender beams while maintaining these 3-DOF. This configuration ensures that translation and rotation motions remain insensitive to axial loads (Figure 2b) while achieving uniform stress distribution. The rotational drive employs piezoelectric ceramics and a parallel four-bar linkage mechanism, converting coaxial force into torque based on deformation principles (Figure 4), thereby improving overall stiffness and load-bearing performance while ensuring motion accuracy.

Owing to the serial configuration of the translation and rotating stages, the XYθ_z_ nano-positioning stage can achieve independent motion in the X, Y, and θ_z_ directions.

## 3. Modelling of XYθ_z_ Stage 

In this paper, the XYθ_z_ three-degree-of-freedom stage is analysed through dynamic modelling, and the static equilibrium equation and geometric constraint equation of the XYθ_z_ driving mechanism are established from the deformation of the flexible hinge and the rigid body and the mechanism motion chain group, respectively. The guiding stiffness of the XYθ_z_ three-degree-of-freedom stage is analysed by means of a compliance matrix, and the motion statics model of the flexible hinge mechanism is realised, and the flexible hinge mechanism of the micro-positioning stage is fully flexibly modelled.

### 3.1. Compliance Model of Flexible Hinge

**Figure 5:**
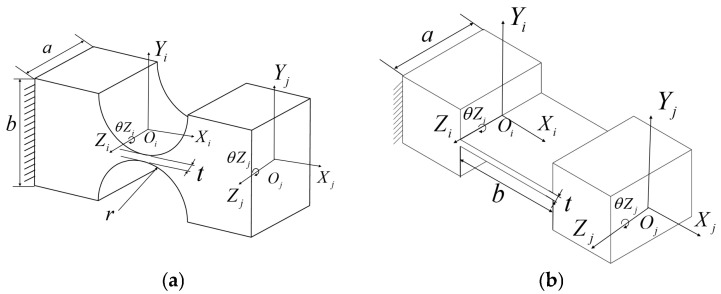
Flexible hinge in the local coordinate system: (**a**) arc-shaped flexible hinge and (**b**) straight beam-type flexible hinge.

The XY (θ_z_) drive unit phase comprises a straight-beam hinge and an arc-shaped hinge. In the local coordinate system, when the load applied to the end of the flexible hinge is $F^{*}={\left[F_{X}^{*},F_{Y}^{*},M^{*}\right]}^{T}$, the axial displacement along the X-axis caused by tensile deformation, transverse displacement of the y-axis, and the shear deformation and bending moment caused by the angular displacement of the rotation around the Z-axis are caused by the axial displacement along the X-axis, transverse displacement of the Y-axis, and angular displacement of rotation around the z-axis (Figure 5). The compliance matrix models and their rotational transformation matrix models for both straight-beam and circular-arc flexure hinges were obtained based on the study by Zhou et al. [19].

(1) $${\bar{X}}_{i}^{*}=C_{i}^{*}F^{*},$$

where $C_{i}^{*}\left(i=1,2\right)$ represents the compliance matrix of arc-shaped and straight beam-shaped flexible hinges in the plane of the local coordinate system.

(2) $$C_{1}^{*=}\left[\begin{array}{ccc} c_{11} & 0 & 0\\ 0 & c_{22} & c_{23}\\ 0 & c_{32} & c_{33} \end{array}\right].$$

The elements in the matrix are

(3) $$c_{11}=\frac{1}{Ea}N_{1},$$

(4) $$c_{22}=\frac{k}{Gr}N_{1}+\frac{12}{Ea}N_{2}-\frac{12}{Ea}N_{3},$$

(5) $$c_{23}=c_{32}=0,$$

(6) $$c_{33}=\frac{12}{Ear^{2}}N_{2},$$

where E is the Young’s modulus of the material, a denotes the width of the hinge, and r denotes the radius of the arc of the hinge. See Appendix A for specific expressions.

(7) $$C_{2}^{*=}\left[\begin{array}{ccc} c_{11} & 0 & 0\\ 0 & c_{22} & c_{23}\\ 0 & c_{32} & c_{33} \end{array}\right].$$

The elements in the matrix are

(8) $$C_{11}=\frac{b}{Eat}.$$

(9) $$C_{22}=\frac{4b^{3}}{3Eat^{3}}+\frac{b}{Gat}.$$

(10) $$C_{23}=\frac{6b^{2}}{Eat^{2}}.$$

(11) $$C_{32}=\frac{6b^{2}}{Eat^{2}}.$$

(12) $$C_{33}=\frac{12b}{Eat^{3}}.$$

### 3.2. Deformation Relationship Between Flexible Hinge and Rigid Rod

If the angle between the flexible hinge and forward direction of the X-axis of the overall coordinate system is α, when the mechanism moves, the flexible hinge will rotate together with the rigid rods connected in series Δθ, and the angle between the flexible hinge and X-axis is (Δθ+α). Therefore, the compliance matrix C in the overall coordinate system has the following relationship with C* in the local coordinate system:

(13) $${C=R^{T}C_{i}}^{*}R\left(i=1,2\right),$$

The transformation matrix R is

(14) $$R=\left[\begin{array}{ccc} \cos\left(\alpha +\Delta \theta \right) & \sin\left(\alpha +\Delta \theta \right) & 0\\ -\sin\left(\alpha +\Delta \theta \right) & \cos\left(\alpha +\Delta \theta \right) & 0\\ 0 & 0 & 1 \end{array}\right].$$

Within the entire XY translation and amplification mechanism, only the flexible hinge undergoes reversible elastic deformation, while the rigid rod remains undeformed, serving solely to transmit load and displacement. The deformation relationship between the flexure hinge and the rigid body was obtained from Chen et al. [20]. The deformation of the flexible hinge and the movement of the flexible hinge–rigid rod combination are analysed below. In the flexible hinge–rigid rod unit, the initial angle between the rigid rod and flexible hinge is $\theta_{i}$,${\;\alpha }_{j}$. Under the action of the terminal load $F=\left[F_{x},F_{y},M\right]$, the rigid rod will not undergo elastic deformation and will only rotate or flatten. When the rigid rod turns by an angle $\Delta \theta_{i}$, the coordinates and displacement of the rigid rod in the x and y directions are

(15) $$U_{i}=\left[\begin{array}{c} u_{xi}\\ u_{yi} \end{array}\right]=d_{i}\left[\begin{array}{c} \cos(\theta_{i}+∆\theta_{i})\\ s\mathrm{in}(\theta_{i}+∆\theta_{i}) \end{array}\right],$$

(16) $$\;∆U_{i}=\left[\begin{array}{c} {∆u}_{xi}\\ {∆u}_{yi} \end{array}\right]=d_{i}\left[\begin{array}{c} \cos(\theta_{i}+∆\theta_{i})\\ s\mathrm{in}(\theta_{i}+∆\theta_{i}) \end{array}\right]-d_{i}\left[\begin{array}{c} \cos(\theta_{i})\\ s\mathrm{in}(\theta_{i}) \end{array}\right],$$

where $d_{i}$ indicates the length of the rigid rod.

**Figure 6:**
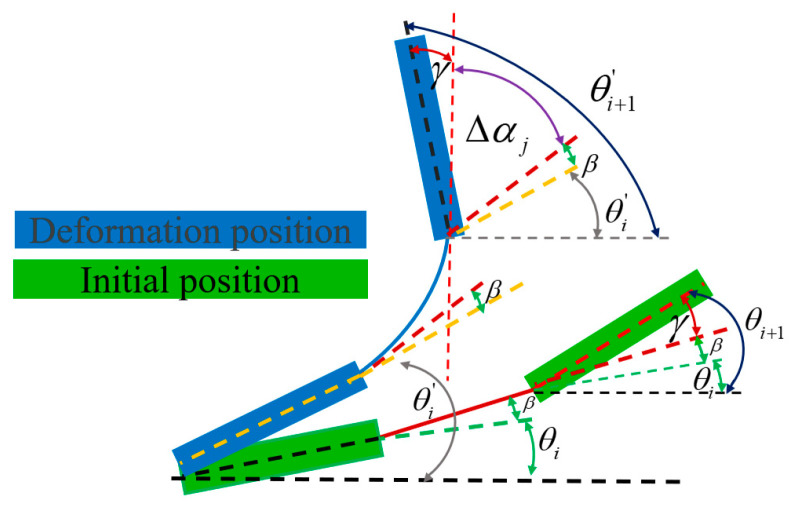
Angle–displacement relationship.

The deformation of the flexible hinge contains two components (Figure 6). The first is the angular displacement of the preceding rigid rod $\left(\Delta \theta_{i}\right)$ resulting from rigid-body displacement. The second is the elastic displacement of the flexible hinge, which includes linear displacements $\Delta X_{i}$ and $\Delta Y_{i}$ along the X- and Y-axes, respectively, in the global coordinate system, as well as angular displacement $\alpha_{j}$ around the vertical z-axis. The coordinates and displacements of the flexible hinge in the x and y directions, after deformation, are given by

(17) $$V_{j}=\left[\begin{array}{c} v_{xi}\\ v_{yi} \end{array}\right]=L_{j}\left[\begin{array}{c} \cos(\alpha_{i}+∆\theta_{i})\\ s\mathrm{in}(\alpha_{i}+∆\theta_{i}) \end{array}\right]+\left[\begin{array}{c} ∆x_{j}\\ {∆x}_{j} \end{array}\right],$$

(18) $$∆V_{j}=\left[\begin{array}{c} {∆v}_{xi}\\ {∆v}_{yi} \end{array}\right]=L_{j}\left[\begin{array}{c} \cos(\alpha_{i}+∆\theta_{i})\\ s\mathrm{in}(\alpha_{i}+∆\theta_{i}) \end{array}\right]-L_{j}\left[\begin{array}{c} \cos(\alpha_{i})\\ s\mathrm{in}(\alpha_{i}) \end{array}\right]+\left[\begin{array}{c} ∆x_{j}\\ {∆x}_{j} \end{array}\right],$$

where $L_{j}$ represents the length of the flexible hinge.

The angular displacement of the rigid rod i+1 indicates the sum of the angular displacement of the adjacent rigid rod i and the associated flexible hinge j (Figure 6):

(19) $$\Delta \theta_{i+1}=\Delta \theta_{i}+\Delta \alpha_{j}.$$

### 3.3. Modelling of the XY Stage

#### 3.3.1. Hybrid Amplification Mechanism

As the hybrid amplification mechanism is symmetric about its axis, only half of the X/Y hybrid amplification mechanism requires analysis.

**Figure 7:**
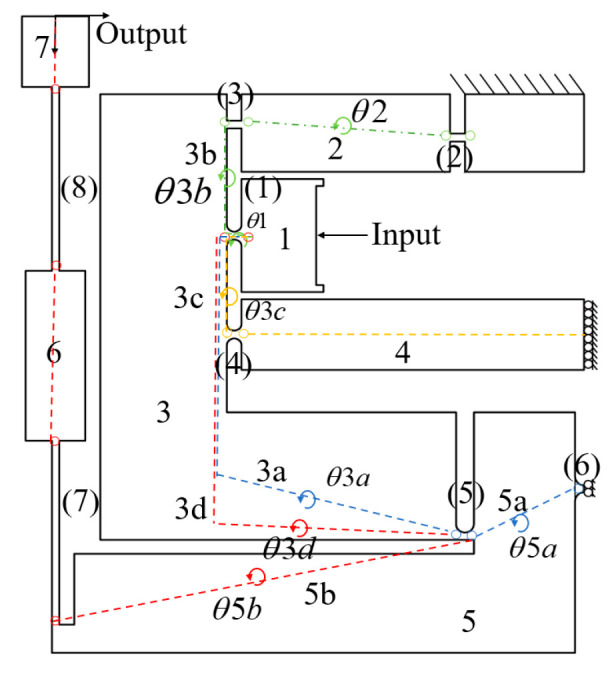
Four motion chains of the XY hybrid amplification mechanism.

The kinematic relationship between the motion transmission chains in the mechanism was characterised based on the study by Chen et al. [20]. The motion transmission chain is decomposed into multiple segments from the input terminal to the output terminal and fixed terminals to establish the geometric relationship between input and output displacements. The displacement of the rigid rods and flexible hinges along the same path is computed, and the corresponding geometric constraints for each segment are determined. These constraints are critical in defining the overall displacement transformation of the mechanism. The XY hybrid amplification mechanism consists of four kinematic chains, each represented by different colors. Rigid links are denoted by "*i*" while flexible hinges are marked by "(*i*)", as shown in Figure 7.

The first motion chain over-constrained equation (1–(1)–3b–(3)–2–(2)):

(20) $$\Delta u_{x1}+\Delta v_{x1}+\Delta u_{x3b}+\Delta v_{x3}+\Delta u_{x2}+\Delta v_{x2}+X_{I_{x}}=0,$$

(21) $$\Delta u_{y1}+\Delta v_{y1}+\Delta u_{y3b}+\Delta v_{y3}+\Delta u_{y2}+\Delta v_{y2}+Y_{I_{x}}=0,$$

The second motion chain over-constrained equation (1–(1)–3c–(4)–4):

(22) $$\Delta u_{x1}+\Delta v_{x1}+\Delta u_{x3c}+\Delta v_{x4}+\Delta u_{x4}+X_{I_{x}}=0,$$

(23) $$\Delta u_{y1}+\Delta v_{y1}+\Delta u_{y3c}+\Delta v_{y4}+\Delta u_{y4}+Y_{I_{x}}=0,$$

The third motion chain over-constrained equation (1–(1)–3a–(5)–5a–(6)):

(24) $$\Delta u_{x1}+\Delta v_{x1}+\Delta u_{x3a}+\Delta v_{x5}+\Delta u_{x5a}+\Delta v_{x6}+X_{I_{x}}=0,$$

(25) $$\Delta u_{y1}+\Delta v_{y1}+\Delta u_{y3a}+\Delta v_{y5}+\Delta u_{y5a}+\Delta v_{y6}+Y_{I_{x}}=0,$$

The fourth motion chain over-constrained equation (1–(1)–3d–(5)–5b–(7)–6–7):

(26) $$\Delta u_{x1}+\Delta v_{x1}+\Delta u_{x3d}+\Delta v_{x5}+\Delta u_{x5b}+\Delta v_{x7}+\Delta u_{x6}+\Delta v_{x8}+\Delta u_{x7}+X_{I_{x}}=X_{out_{x}},$$

(27) $$\Delta u_{y1}+\Delta v_{y1}+\Delta u_{y3d}+\Delta v_{y5}+\Delta u_{y5b}+\Delta v_{y7}+\Delta u_{y6}+\Delta v_{y8}+\Delta u_{y7}+Y_{I_{x}}=Y_{out_{x}},$$

From Equation (19), the geometric relationship between the rigid rod and flexible hinge in the XY amplification mechanism is expressed as

(28) $$\Delta \theta_{1}+\Delta \alpha_{1}=\Delta \theta_{3},$$

(29) $$\Delta \theta_{2}+\Delta \alpha_{2}=0,$$

(30) $$\Delta \theta_{3}+\Delta \alpha_{3}=\Delta \theta_{2},$$

(31) $$\Delta \theta_{3}+\Delta \alpha_{4}=\Delta \theta_{4},$$

(32) $$\Delta \theta_{3}+\Delta \alpha_{5}=\Delta \theta_{5},$$

(33) $$\Delta \theta_{5}+\Delta \alpha_{6}=0,$$

(34) $$\Delta \theta_{6}+\Delta \alpha_{8}=\Delta \theta_{7},$$

(35) $$\Delta \theta_{5}+\Delta \alpha_{7}=\Delta \theta_{6}.$$

**Figure 8:**
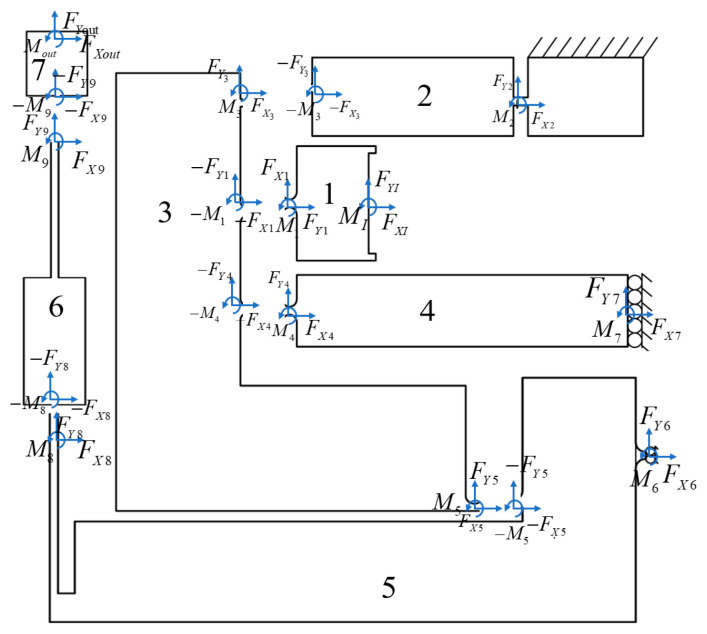
Free-body diagram of XY hybrid amplification mechanism.

The geometric equations of the XY hybrid amplification mechanism have been derived from the above analysis. Subsequently, a force analysis should be performed to complete the static model of the XY hybrid amplification mechanism (Figure 8).

Static equilibrium equations of rigid rod 1:

(36) $$F_{X_{1}}+F_{X_{I}}=0,$$

(37) $$F_{Y_{1}}+F_{Y_{I}}=0,$$

(38) $$M_{1}+M_{I}+F_{X_{I}}\left(u_{x_{1}}+v_{x_{1}}\right)+F_{Y_{I}}\left(u_{y_{1}}+v_{y_{1}}\right)=0,$$

Static equilibrium equations of rigid rod 2:

(39) $$F_{X_{2}}-F_{X_{3}}=0,$$

(40) $$F_{Y_{2}}-F_{Y_{3}}=0,$$

(41) $$M_{2}-M_{3}+F_{X_{2}}\left(u_{x_{2}}+v_{x_{2}}\right)+F_{Y_{2}}\left(u_{y_{2}}+v_{y_{2}}\right)=0.$$

Static equilibrium equations of rigid rod 3:

(42) $$F_{X_{3}}-F_{X_{4}}-F_{X_{1}}+F_{X_{5}}=0,$$

(43) $$F_{Y_{3}}-F_{Y_{4}}-F_{Y_{1}}+F_{Y_{5}}=0,$$

(44) $$M_{5}+M_{3}-M_{1}-M_{4}+F_{X_{5}}\left(u_{x_{3a}}+v_{x_{5}}\right)+F_{Y_{5}}\left(u_{y_{3a}}+v_{y_{5}}\right)+F_{X_{3}}\left(u_{x_{3b}}+v_{x_{3}}\right)\;+F_{Y_{3}}\left(u_{y_{3b}}+v_{y_{3}}\right)-F_{Y_{4}}\left(u_{y_{3c}}+v_{y_{3}}\right)-F_{X_{4}}\left(u_{x_{3c}}+v_{x_{3}}\right)=0.$$

Static equilibrium equations of rigid rod 4:

(45) $$F_{X_{4}}+F_{X_{7}}=0,$$

(46) $$F_{Y_{4}}+F_{Y_{7}}=0,$$

(47) $$M_{4}+M_{7}+F_{X_{4}}\left(u_{x_{4}}+v_{x_{4}}\right)+F_{Y_{4}}\left(u_{y_{4}}+v_{y_{4}}\right)=0.$$

Static equilibrium equations of rigid rod 5:

(48) $$F_{X_{6}}-F_{X_{8}}-F_{X_{5}}=0,$$

(49) $$F_{Y_{6}}-F_{Y_{8}}-F_{Y_{5}}=0,$$

(50) $$M_{6}-M_{8}-M_{5}+F_{X_{6}}\left(u_{x_{5a}}+v_{x_{6}}\right)+F_{Y_{6}}\left(u_{y_{5a}}+v_{y_{6}}\right)+F_{X_{8}}\left(u_{x_{5b}}+v_{x_{7}}\right)+F_{Y_{8}}\left(u_{y_{5b}}+v_{y_{7}}\right)=0,$$

Static equilibrium equations of rigid rod 6:

(51) $$F_{X_{9}}-F_{X_{8}}=0,$$

(52) $$F_{Y_{9}}-F_{Y_{8}}=0,$$

(53) $$M_{9}-M_{8}+F_{X_{9}}\left(u_{x_{6}}+v_{x_{8}}\right)+F_{Y_{9}}\left(u_{y_{6}}+v_{y_{8}}\right)=0.$$

Static equilibrium equations of rigid rod 7:

(54) $$F_{X_{out}}-F_{X_{9}}=0,$$

(55) $$F_{Y_{out}}-F_{Y_{9}}=0,$$

(56) $$M_{out}-M_{9}+F_{X_{out}}\left(u_{x_{7}}+X_{outx}\right)+F_{Y_{out}}\left(u_{y_{7}}+Y_{outx}\right)=0.$$

Based on Equations (20)–(56), given the input displacement $X_{I}$ or input force $F_{YI}$, a system of 37 equations with 37 unknowns is established. Under the assumption of zero load at the output end of the XY hybrid compliant mechanism (where the output force equals zero), the output displacements $X_{outx}$ and $Y_{outx}$ can be determined by solving this equation system.

(57) $$\begin{array}{l} X_{outx}=\left(d_{5b}-d_{5a}\right)\left[\cos\left(\Delta \theta_{1}+\Delta \alpha_{1}+\Delta \alpha_{5}\right)-\cos\left(\theta_{5}\right)\right]+L_{7}[\cos(\alpha_{7}+\Delta \theta_{1}+\\ {\Delta \alpha }_{1}+\Delta \alpha_{5})-\cos\left(\alpha_{7}\right)]+d_{6}\left[\cos\left(\theta_{6}+\Delta \theta_{1}+\Delta \alpha_{1}+\Delta \alpha_{5}+\Delta \alpha_{7}\right)-\cos\left(\theta_{6}\right)\right]+\\ L_{8}\left[\cos\left(\theta_{8}+\Delta \theta_{1}+\Delta \alpha_{1}+\Delta \alpha_{5}+\Delta \alpha_{7}\right)-\cos\left(\alpha_{8}\right)\right]+d_{7}[\cos(\theta_{7}+\Delta \theta_{1}+\Delta \alpha_{1}+\\ {\Delta \alpha }_{7}+\Delta \alpha_{8})-\cos\left(\theta_{7}\right)]-L_{6}\left[\cos\left(\alpha_{7}+\Delta \theta_{1}+\Delta \alpha_{1}+\Delta \alpha_{5}\right)-\cos\left(\alpha_{7}\right)\right]-\Delta X_{6}+\\ {\Delta X}_{7}+\Delta X_{8}. \end{array}$$

Here, $∆\alpha_{8}=F_{outx}*{R^{T}C}_{8}R$.

(58) $$[Y_{Ix}=d_{1}\left[\sin\left(\theta_{1}\right)-\sin\left(\theta_{1}+\Delta \theta_{1}\right)\right]+L_{1}\left[\sin\left(\alpha_{1}\right)-\sin\left(\alpha_{1}+\Delta \theta_{1}\right)\right]+d_{3a}\left[\sin\left(\theta_{3}\right)-\sin\left(\theta_{3}+\Delta \theta_{1}+\Delta \alpha_{1}\right)\right]+\;L_{5}\left[\sin\left(\alpha_{5}\right)-\sin\left(\alpha_{5}+\Delta \theta_{1}+\Delta \alpha_{1}\right)\right]+d_{5a}\left[\sin\left(\theta_{7}\right)-\sin\left(\theta_{5}+\Delta \theta_{1}+\Delta \alpha_{1}+\Delta \alpha_{5}\right)\right]+L_{6}\left[\sin\left(\alpha_{6}\right)-\sin\left(\alpha_{6}+\;{\Delta \theta }_{1}+\Delta \alpha_{1}+\Delta \alpha_{5}\right)\right]\pm \left(\Delta Y_{1}+\Delta Y_{5}+\Delta Y_{6}\right)].$$

#### 3.3.2. Planner Guiding the Mechanism of XY Stage

**Figure 9:**
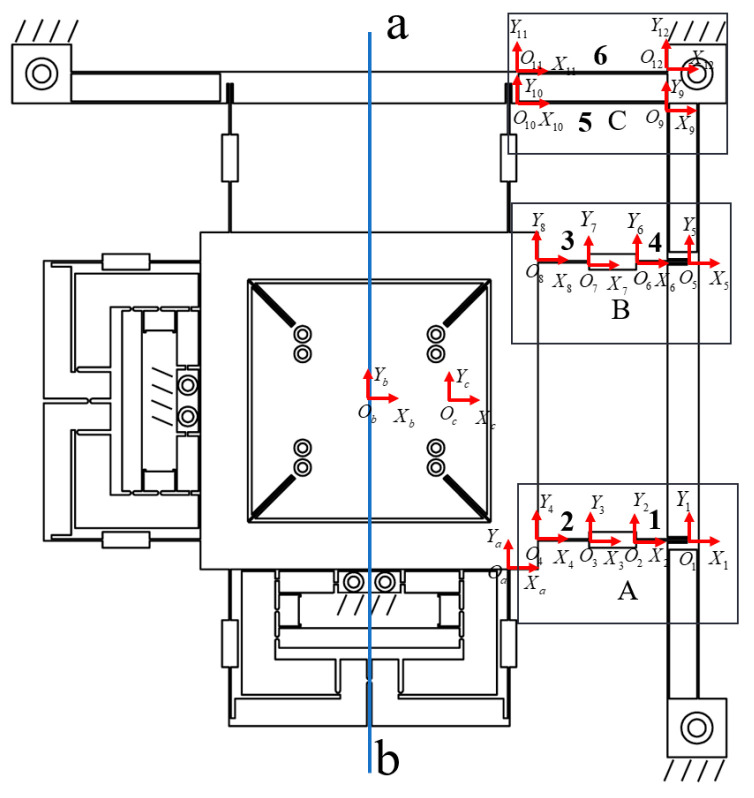
Flexible guide mechanism of the XY stage and hinge division diagram.

**Figure 10:**
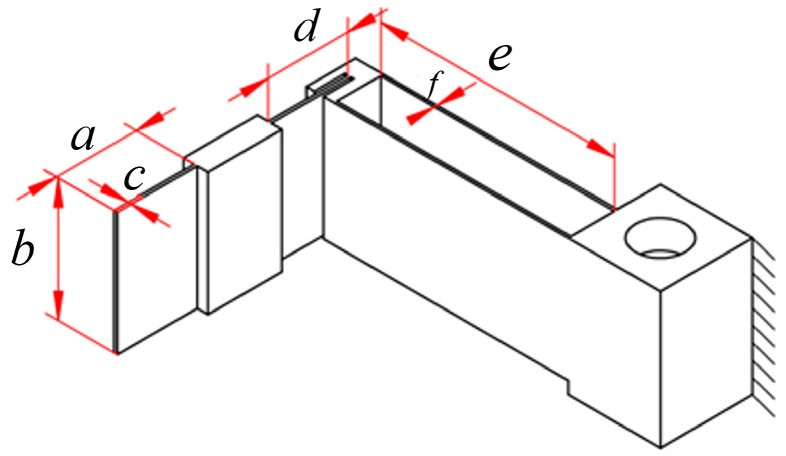
Size of the guide mechanism of part of the XY stage.

The guide mechanism is divided into two sections for compliance matrix modelling—modelling the XY plane guide mechanism and modelling the guide mechanism of the XY stage through the over-constrained mechanism and guide mechanism of θ_z_. The constraint mechanism also functions as part of the XY stage guide system. As the XY stage is symmetrical about the axis of linear *ab* rotation, only half of the XY guide mechanism requires modelling and analysis. The XY plane guide mechanism can be represented as three parallel modules (A, B, and C) (Figure 9). Figure 10 shows the dimensioning of part of the guide mechanism of the XY stage, and Table 2 presents the specific parameters of the guide mechanism.

The compliance matrix from hinge 1 to output terminal $O_{a}$ is

(59) $$C_{1}=C_{O_{1}}^{O_{a}}+C_{O_{2}}^{O_{a}}.$$

Module A consists of hinges 1 and 2 connected in series. According to the serial–parallel matrix theory for flexure hinge mechanisms established by Koseki et al. [21], the overall compliance matrix of this module in the global coordinate system can be expressed as:

(60) $$C_{A}=C_{O_{1}}^{O_{a}}+C_{O_{2}}^{O_{a}}+C_{O_{3}}^{O_{a}}+C_{O_{4}}^{O_{a}}.$$

From Equation (32):

(61) $$C_{A}=T_{O_{1}}^{O_{a}}C_{O_{1}}{\left(T_{O_{1}}^{O_{a}}\right)}^{T}+T_{O_{2}}^{O_{a}}C_{O_{2}}{\left(T_{O_{2}}^{O_{a}}\right)}^{T}+T_{O_{3}}^{O_{a}}C_{O_{3}}{\left(T_{O_{3}}^{O_{a}}\right)}^{T}+T_{O_{4}}^{O_{a}}C_{O_{4}}{\left(T_{O_{4}}^{O_{a}}\right)}^{T}.$$

Module B is composed of 3 and 4 in series; its overall compliance matrix in global coordinates is

(62) $$C_{B}=T_{O_{5}}^{O_{a}}C_{O_{5}}{\left(T_{O_{5}}^{O_{a}}\right)}^{T}+T_{O_{6}}^{O_{a}}C_{O_{6}}{\left(T_{O_{6}}^{O_{a}}\right)}^{T}+T_{O_{7}}^{O_{a}}C_{O_{7}}{\left(T_{O_{7}}^{O_{a}}\right)}^{T}+T_{O_{8}}^{O_{a}}C_{O_{8}}{\left(T_{O_{8}}^{O_{a}}\right)}^{T}.$$

Module C is composed of 5 and 6 in parallel; its overall compliance matrix in global coordinates is

(63) $$\;C_{C}={\left({\left(T_{O_{9}}^{O_{a}}C_{O_{9}}{\left(T_{O_{9}}^{O_{a}}\right)}^{T}+T_{O_{10}}^{O_{a}}C_{O_{10}}{\left(T_{O_{10}}^{O_{a}}\right)}^{T}\right)}^{-1}+{\left(T_{O_{11}}^{O_{a}}C_{O_{11}}{\left(T_{O_{11}}^{O_{a}}\right)}^{T}+T_{O_{12}}^{O_{a}}C_{O_{12}}{\left(T_{O_{12}}^{O_{a}}\right)}^{T}\right)}^{-1}\right)}^{-1}.$$

Modules A, B, and C are connected in parallel in the global coordinate system; the output compliance matrix in global coordinates $O_{a}$ is

(64) $$C_{outya}{=\left(C_{A}^{-1}+C_{B}^{-1}+C_{C}^{-1}\right)}^{-1}.$$

#### 3.3.3. Over-Constrained Guiding Mechanism

**Figure 11:**
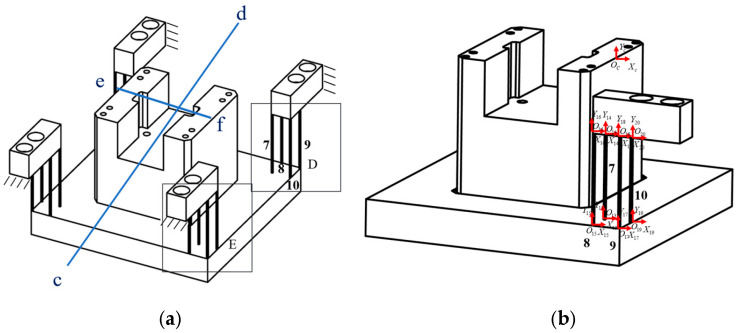
Modelling system of over-constrained mechanism guide mechanism: (**a**) hinge division diagram of the over-constrained mechanism guide mechanism; (**b**) local coordinate map of the hinge of the guide mechanism through the over-constrained mechanism.

**Figure 12:**
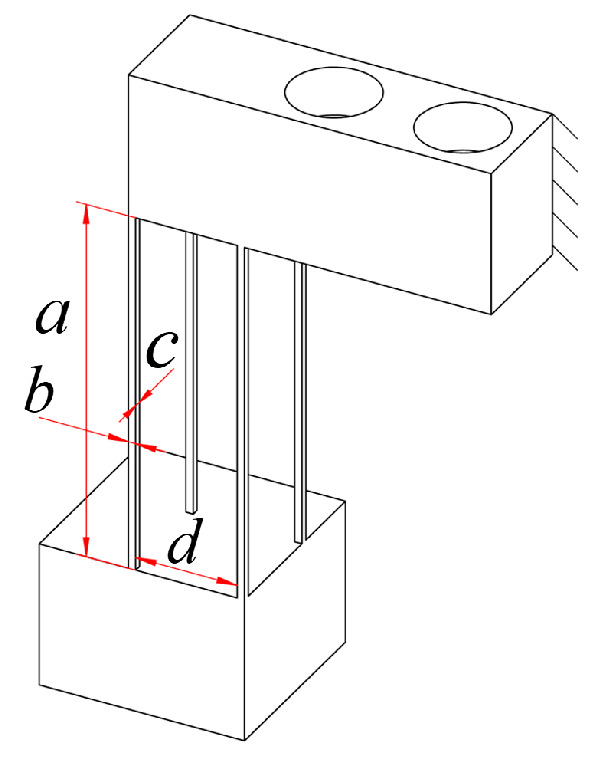
Size of the guide mechanism of part of the over-constrained mechanism.

The over-constrained mechanisms are symmetrical about the *cd* axis; only one-half requires analysis, which can be represented as modules D and E in parallel. As modules D and E are symmetrical about the *ef*-axis (Figure 11), only module D is modelled and analysed. Figure 12 shows part of the guiding mechanism of the over-constrained mechanism, with specific dimensions provided in Table 3.

Module D is composed of 7–10 in parallel; its compliance matrix in the global coordinate system $O_{c}$ is

(65) $$\begin{array}{ll} C_{D} & ={\left(T_{O_{13}}^{O_{b}}C_{O_{13}}{\left(T_{O_{13}}^{O_{b}}\right)}^{T}+T_{O_{14}}^{O_{b}}C_{14}{\left(T_{O_{14}}^{O_{b}}\right)}^{T}\right)}^{-1}+{\left(T_{O_{15}}^{O_{b}}C_{O_{15}}{\left(T_{O_{15}}^{O_{b}}\right)}^{T}+T_{O_{16}}^{O_{b}}C_{16}{\left(T_{O_{16}}^{O_{b}}\right)}^{T}\right)}^{-1}\\ & +{\left(T_{O_{17}}^{O_{b}}C_{O_{17}}{\left(T_{O_{17}}^{O_{b}}\right)}^{T}+T_{O_{18}}^{O_{b}}C_{18}{\left(T_{O_{18}}^{O_{b}}\right)}^{T}\right)}^{-1}+{\left(T_{O_{19}}^{O_{b}}C_{O_{19}}{\left(T_{O_{19}}^{O_{b}}\right)}^{T}+T_{O_{20}}^{O_{b}}C_{20}{\left(T_{O_{20}}^{O_{b}}\right)}^{T}\right)}^{-1}. \end{array}$$

Modules D and E are connected in parallel under in the local coordinate system $O_{b}$ of the guide mechanism; the output compliance matrix in the global coordinate system is

(66) $$C_{O_{C}}={\left(C_{D}^{-1}+C_{E}^{-1}\right)}^{-1}.$$

The guide mechanism of the over-constrained mechanism is symmetrical about the straight line *cd*, $C_{O_{C}}\;$ is transformed by the coordinates, and the compliance matrix of the over-constrained mechanism $C_{O_{C2}}$ is

(67) $$C_{O_{C2}}=T_{1}C_{O_{C}}T_{1}^{T}.$$

$C_{O_{C}}$,$C_{O_{C2}}$ is connected in parallel; the compliance matrix of the over-constrained mechanism is passed, and then the compliance matrix of the over-constrained guide mechanism in the global coordinate $O_{b}$ is

(68) $$C_{outb}={\left(C_{O_{C}}^{-1}+C_{O_{C2}}^{-1}\right)}^{-1}.$$

#### 3.3.4. Overall XY Stage

As the XY hybrid amplification mechanism contains two output points, one output point is analysed. As the XY stage is moving, the guide mechanism of the over-constrained mechanism is involved in the motion guidance of the XY axis and is connected in parallel with the XY stage; the compliance matrix of the guide mechanism when the XY stage is moving along the X axis is

(69) $$C_{outx}={\left({\left(C_{outya}\right)}^{-1}+{\left(T_{O_{c}}^{O_{a}}C_{outb}{\left(T_{O_{c}}^{O_{a}}\right)}^{T}\right)}^{-1}\right)}^{-1}.$$

The input/output displacements of the X/Y hybrid amplification mechanism can be determined according to Equations (26) and (27). In the XY stage, when the output end of the X/Y hybrid amplification mechanism possesses guiding stiffness, the output force *F* is given by

(70) $$F_{outx}=C_{outx}\times {\bar{X}}_{X_{outx}}.$$

where ${\bar{X}}_{X_{outx}}$ represents the output displacement matrix in both X and Y directions of the X-axis hybrid motion-amplifying mechanism.

The output force $F_{outx}$ of the XY hybrid amplification mechanism is incorporated into both the equilibrium equations and geometric Equations (13)–(24) of the XY hybrid amplification mechanism. Since the output force $F_{outx}$ is a dependent variable on the output displacement, the number of equations remains equal to the number of unknowns, ensuring that the XY stage has a unique solution $X_{x}$. The magnification ratio of the X-axis in the XY stage can be calculated using the following formula:

The output force $F_{outx}$ of the XY hybrid motion-amplifying mechanism is introduced into both its equilibrium and geometric equations. As the output force $F_{outx}$ is functionally dependent on the output displacement $X_{x}$, the number of equations consistently matches the number of unknowns. When either the input force $F_{YI}$ or input displacement $Y_{I}$ is specified, the output displacement of the XY stage can be uniquely determined. The motion amplification ratio along the X-axis can be calculated using the following expression:

(71) $$\lambda_{X}=\frac{X_{x}}{Y_{I}}.$$

Equivalent output stiffness of the XY stage along the X-axis:

(72) $$K_{outx}=\frac{F_{outx}}{X_{x}}.$$

The magnification ratio and output stiffness of the X-axis in the XY stage can be derived analogously to those of the Y-axis, following the same theoretical framework.

### 3.4. Modelling of the θ_z_ Stage

**Figure 13:**
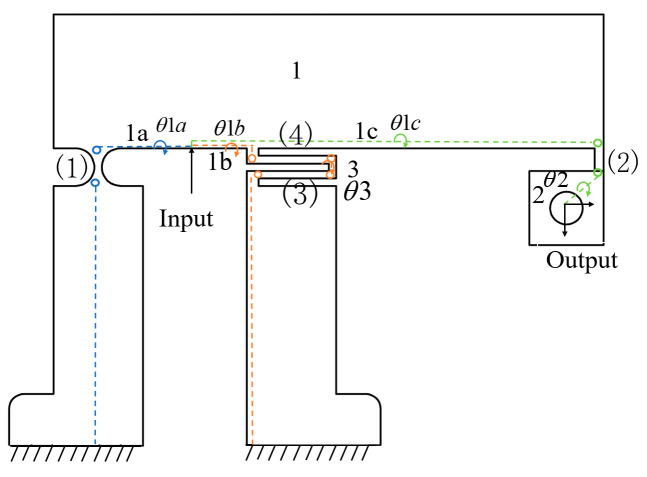
Three motion chains driven by the θ_z_ amplification mechanism.

As the θ_z_ amplification mechanism contains two symmetrically distributed lever displacement amplification mechanisms, only half of the unit requires analysis. The θ_z_ amplification mechanism consists of four kinematic chains distinguished by different colors, where rigid links are labeled "i" and flexible hinges are marked "(i)", as shown in Figure 13.

#### Geometric Constraints of the θ_z_ Amplification Mechanism

The θ_z_ amplification mechanism has three motion chains, and the over-constrained equation of the first motion chain (1a–(1)) is

(73) $$\Delta u_{x1a}+\Delta v_{x1}+X_{I_{\theta Z}}=0,$$

(74) $$\Delta u_{y1a}+\Delta v_{y1}+Y_{I_{\theta Z}}=0.$$

The constrained equation of the second motion chain (1b–(4)–3–(3)) is

(75) $$\Delta u_{x1b}+\Delta v_{x4}+\Delta v_{x3}+\Delta u_{x3}+X_{I_{\theta Z}}=0,$$

(76) $$\Delta u_{y1b}+\Delta v_{y4}+\Delta v_{y3}+\Delta u_{y3}+Y_{I_{\theta Z}}=0.$$

The constrained equation of the third motion chain (1c–(2)–2) is

(77) $$\Delta u_{x1c}+\Delta v_{x2}+\Delta u_{x2}+X_{I_{\theta Z}}=X_{out_{\theta Z}},$$

(78) $$\Delta u_{y1c}+\Delta v_{y2}+\Delta u_{y2}+Y_{I_{\theta Z}}=Y_{out_{\theta Z}}.$$

From Equation (12), the geometric relationship between the rigid rod and the flexible hinge in the θ_z_ amplification mechanism is obtained as

(79) $$\Delta \theta_{1}+\Delta \alpha_{1}=0,$$

(80) $$\Delta \theta_{1}+\Delta \alpha_{4}=\Delta \theta_{3},$$

(81) $$\Delta \theta_{1}+\Delta \alpha_{2}=\Delta \theta_{2},$$

(82) $$\Delta \theta_{3}+\Delta \alpha_{3}=0.$$

**Figure 14:**
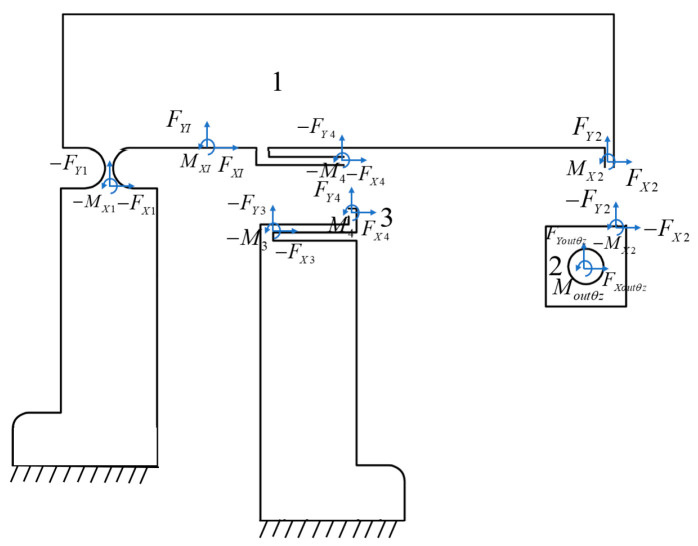
Free-body diagram of the θ_z_ amplification mechanism.

The geometric equations of the θ_z_ amplification mechanism have been derived from the above analysis. Subsequently, a force analysis should be performed to complete the static model of the θ_z_ amplification mechanism (Figure 14).

Static equilibrium equations of rigid rod 1:

(83) $$F_{X_{2}}-F_{X_{4}}-F_{X_{1}}+F_{X_{I}}=0,$$

(84) $$F_{Y_{2}}-F_{Y_{4}}-F_{Y_{1}}+F_{Y_{I}}=0,$$

(85) $$M_{2}-M_{1}+M_{I}-M_{4}-F_{X_{1}}\left(u_{x_{2a}}+v_{x_{1}}\right)+F_{X_{2}}\left(u_{x_{2c}}+v_{x_{1}}\right)-F_{X_{4}}\left(u_{x_{2b}}+v_{x_{4}}\right)+\;F_{Y_{2}}\left(u_{y_{2c}}+v_{y_{2}}\right)-F_{Y_{1}}\left(u_{y_{2a}}+v_{y_{1}}\right)-F_{Y_{4}}\left(u_{y_{2b}}+v_{y_{4}}\right)=0.$$

Static equilibrium equations of rigid rod 2:

(86) $$-F_{X_{2}}+F_{X_{out\theta z}}=0,$$

(87) $$-F_{Y_{2}}+F_{Y_{out\theta z}}=0\;,$$

(88) $$M_{O1}-M_{2}+F_{Y_{out\theta z}}Y_{out_{\theta Z}}+F_{X_{out\theta z}}X_{out_{\theta Z}}=0.$$

Static equilibrium equations of rigid rod 3:

(89) $$-F_{X_{3}}+F_{X_{4}}=0,$$

(90) $$-F_{Y_{3}}+F_{Y_{4}}=0,$$

(91) $$-M_{3}+M_{4}+F_{X_{3}}\left(u_{x_{3}}+v_{x_{3}}\right)+F_{X_{3}}\left(u_{x_{3}}+v_{x_{3}}\right)=0.$$

Based on Equations (73)–(91), given either the input displacement $X_{I}$ or input force $F_{YI}$, a nonlinear system of 18 equations with 18 unknowns can be established. Under the zero-load condition at the output end of the θ_z_ motion-amplifying mechanism (where the output force equals zero), the output displacements $Y_{I\theta z}$ and $Y_{out\theta z}$ can be determined by solving this equation system. The output angular displacement (in radians) of the θ_z_ amplification mechanism can be expressed as

(92) $$\theta =\arctan\left(\frac{Y_{out\theta z}}{d_{1c}}\right),$$

where $d_{1c}$ denotes the length of the rigid rod.

(93) $$\;Y_{I\theta z}=d_{1b}\left[\sin\theta_{1}-\sin\left(\Delta \theta_{1}+\Delta \theta_{1}\right)\right]+L_{4}\left[\sin\alpha_{4}-\sin\left(\alpha_{4}+\Delta \theta_{1}\right)\right]+\;\;d_{3}\left[\sin\theta_{3}-\sin\left(\theta_{3}+\Delta \theta_{1}+\Delta \alpha_{4}\right)\right]+L_{3}\left[\sin\left(\alpha_{3}+\Delta \theta_{1}+\Delta \alpha_{4}\right)-\sin\left(\alpha_{3}\right)\right]-\left(\Delta Y_{3}+\Delta Y_{4}\right),$$

(94) $$Y_{out\theta z}=d_{1c}\left[\sin\left(\Delta \theta_{1c}+\Delta \alpha_{2}\right)-\sin\left(\theta_{1c}\right)\right]+L_{2}\left[\sin\left(\alpha_{2}+\Delta \theta_{1}\right)-\sin\left(\alpha_{2}\right)\right]+d_{1a}\left[\sin\left(\Delta \theta_{1a}+\Delta \alpha_{1}\right)-\sin\left(\theta_{1a}\right)\right]\;+L_{1}\left[\sin\left(\alpha_{1}+\Delta \theta_{1}\right)-\sin\left(\alpha_{1}\right)\right]+2\Delta Y_{1}.\;$$

**Figure 15:**
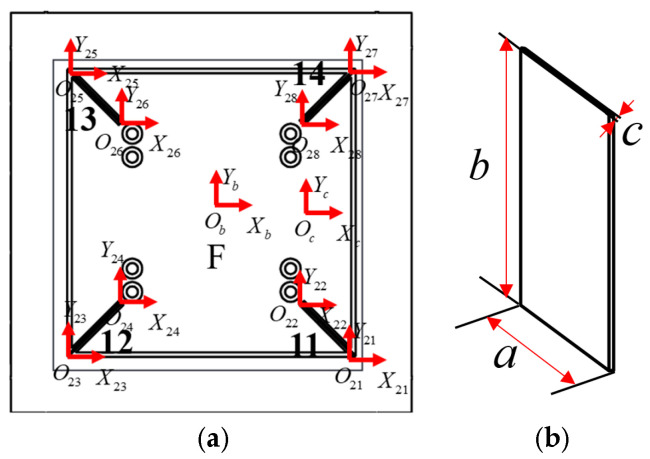
(**a**) Coordinates and distribution map of the hinge of the θ_z_ rotating stage. (**b**) Size of the guide mechanism of part of the θ_z_ stage.

Figure 15 shows the dimensioning of part of the guide mechanism of the θ_z_ stage, and Table 4 presents the specific parameters of the guide mechanism.

Module F is composed of 11–14 in parallel; its compliance matrix in the global coordinate system is

(95) $$C_{F}={\left(T_{O_{21}}^{O_{b}}C_{O_{21}}{\left(T_{O_{21}}^{O_{b}}\right)}^{T}+T_{O_{22}}^{O_{b}}C_{22}{\left(T_{O_{22}}^{O_{b}}\right)}^{T}\right)}^{-1}+{\left(T_{O_{23}}^{O_{b}}C_{O_{23}}{\left(T_{O_{23}}^{O_{b}}\right)}^{T}+T_{O_{24}}^{O_{b}}C_{24}{\left(T_{O_{24}}^{O_{b}}\right)}^{T}\right)}^{-1}\;+{\left(T_{O_{25}}^{O_{b}}C_{O_{25}}{\left(T_{O_{25}}^{O_{b}}\right)}^{T}+T_{O_{26}}^{O_{b}}C_{26}{\left(T_{O_{26}}^{O_{b}}\right)}^{T}\right)}^{-1}{\left(T_{O_{27}}^{O_{b}}C_{O_{27}}{\left(T_{O_{27}}^{O_{b}}\right)}^{T}+T_{O_{28}}^{O_{b}}C_{22}{\left(T_{O_{28}}^{O_{b}}\right)}^{T}\right)}^{-1}.$$

As the over-constrained mechanism during the movement of the θ_z_ axis has a guiding effect on the motion direction, the compliance matrix of the guiding mechanism during motion of the θ_z_ stage is given as

(96) $$C_{out\theta Z}={\left({\left(C_{outyF}\right)}^{-1}+{\left(T_{O_{c}}^{O_{b}}C_{outQ}{\left(T_{O_{c}}^{O_{b}}\right)}^{T}\right)}^{-1}\right)}^{-1}.$$

The input/output displacements of the θ_z_ amplification mechanism can be determined according to Equations (50) and (51). In the θ_z_ stage, when the output end of the θ_z_ amplification mechanism possesses guiding stiffness, the output force $F_{out\theta z}$ is given by

(97) $$F_{out\theta z}=C_{out\theta Z}\times {\bar{X}}_{out\theta Z}.$$

where ${\bar{X}}_{outx\theta Z}$ represents the output displacement matrix in both X and Y directions of the θ_z_ -axis hybrid motion-amplifying mechanism.

The output force $\;F_{out\theta z}$ of the θ_z_ amplification mechanism is introduced into the equilibrium equation and geometric Equations (73)–(91) of the θ_z_ amplification mechanism. Since the output force $F_{out\theta z}$ is the dependent variable on the output displacement, the number of equations remains the same as the unknown number; thus, the θ_z_ stage has a unique solution ${\bar{X}}_{\theta Z}$. The maximum output angle of the θ_z_ stage can be obtained using the following formula:

(98) $$\theta =\arctan\left(\frac{Y_{\theta z}}{d_{1c}}\right).$$

Equivalent output stiffness of the θ_z_ stage.

(99) $$K_{out\theta z}=\frac{F_{out\theta z}}{Y_{\theta z}}.$$

## 4. Finite Element Simulation

Static finite element analysis was conducted using ANSYS software (ANSYS 2022R1) to verify the accuracy of the compliant model for the nano-positioning stage in the flexure-based driving mechanism. The material selected was AL-7075 alloy with a Young’s modulus of 70 GPa and a Poisson’s ratio of 0.33.

### 4.1. Static Analysis Results

**Figure 16:**
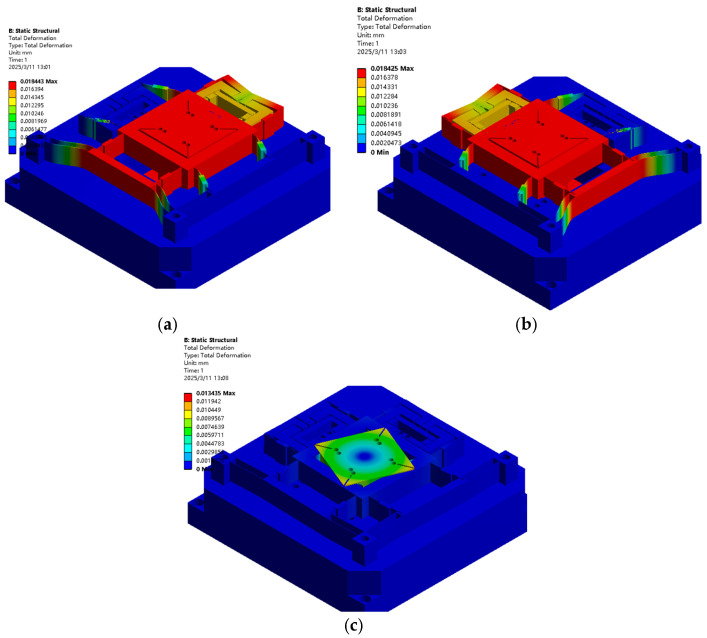
ANSYS finite element simulation results under 100 N equivalent load: (**a**) X-axis deformation; (**b**) Y-axis deformation; and (**c**) θ_z_-axis deformation.

**Figure 17:**
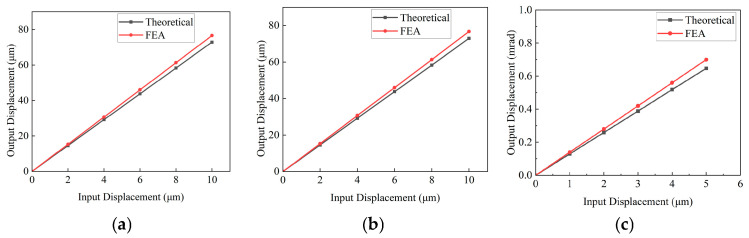
Relationship between the input and output displacement of the finite element analysis and theoretical results: (**a**) X-axis; (**b**) Y-axis; and (**c**) θ_z_-axis.

Constraint conditions were applied at the fixed end, whereas equivalent loads of 100 N were imposed at both input ends of the XYθ_z_ stage driving mechanism to analyse its deformation characteristics, as illustrated in Figure 16. Additionally, displacements of 5 μm were applied at the driving ends to evaluate the displacement amplification ratio of the mechanism, as shown in Figure 17. Refer to Table 5 for the detailed parameters of the X/θ_z_ amplification mechanism.

Figure 16 compares the output displacement of the X-, Y-, and θ_z_-axes under a 100 N equivalent load with the theoretical modelling results. The discrepancy between the FEA values and the theoretical values is 3.43%, 3.34%, and 2.14%, respectively. Additionally, Figure 17 illustrates the relationship between the input and output displacement of the FEA and theoretical results, where the difference between the simulated and theoretical values is 2.77%, 2.7%, and 4.64%, respectively. The comparative analysis is presented in Table 6. The FEA results show certain deviations from theoretical values, primarily due to differences between the simplified assumptions of the theoretical model and the actual physical characteristics [8]. Specifically, the theoretical model typically treats certain components in the mechanism as ideal rigid bodies, whereas FEA can reveal the slight elastic deformations of these components under actual loading conditions. Furthermore, theoretical calculations often neglect practical factors such as material nonlinearity and assembly clearances, whereas FEA simulations, through precise contact algorithms and material constitutive models, can more comprehensively account for these complex characteristics, leading to discrepancies in the stiffness evaluation and deformation prediction obtained using the two methods.

**Figure 18:**
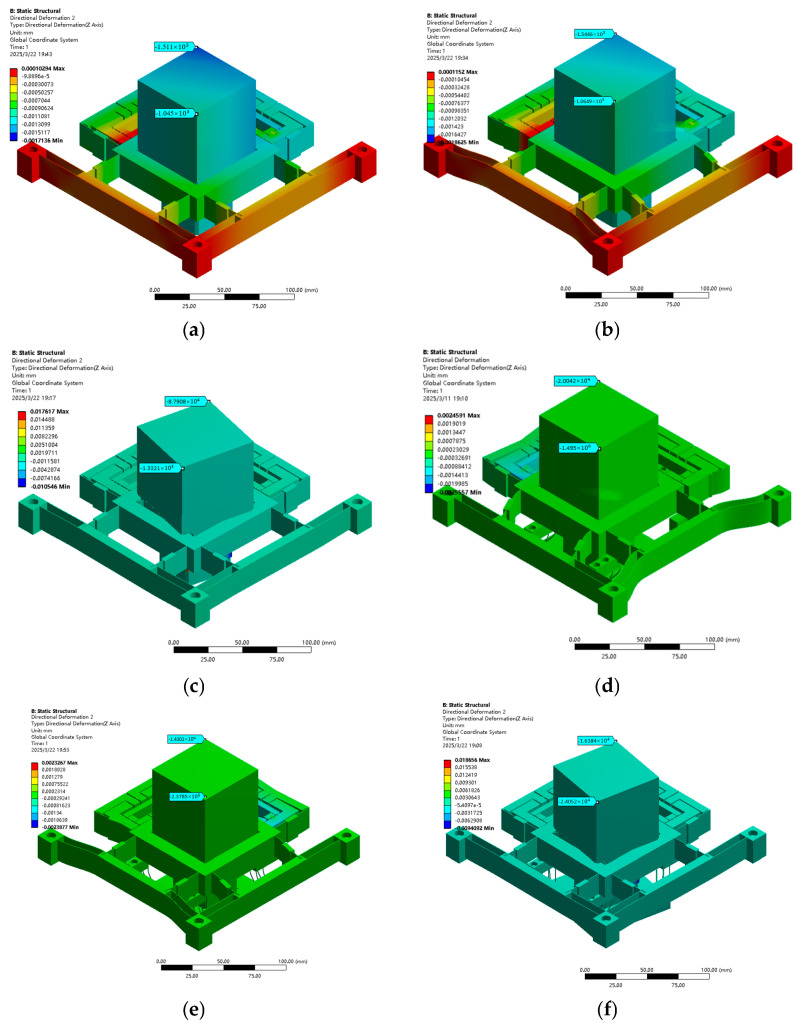
Flatness of FEA load bearing 4KG with or without over-constrained mechanism: (**a**) X-axis without over-constrained mechanism, (**b**) X-axis with over-constrained mechanism (**c**) Y-axis without over-constrained mechanism, (**d**) Y- axis with over-constrained mechanism (**e**) θ_z_-axis without over-constrained mechanism, and (**f**) θ_z_-axis with over-constrained mechanism.

**Figure 19:**
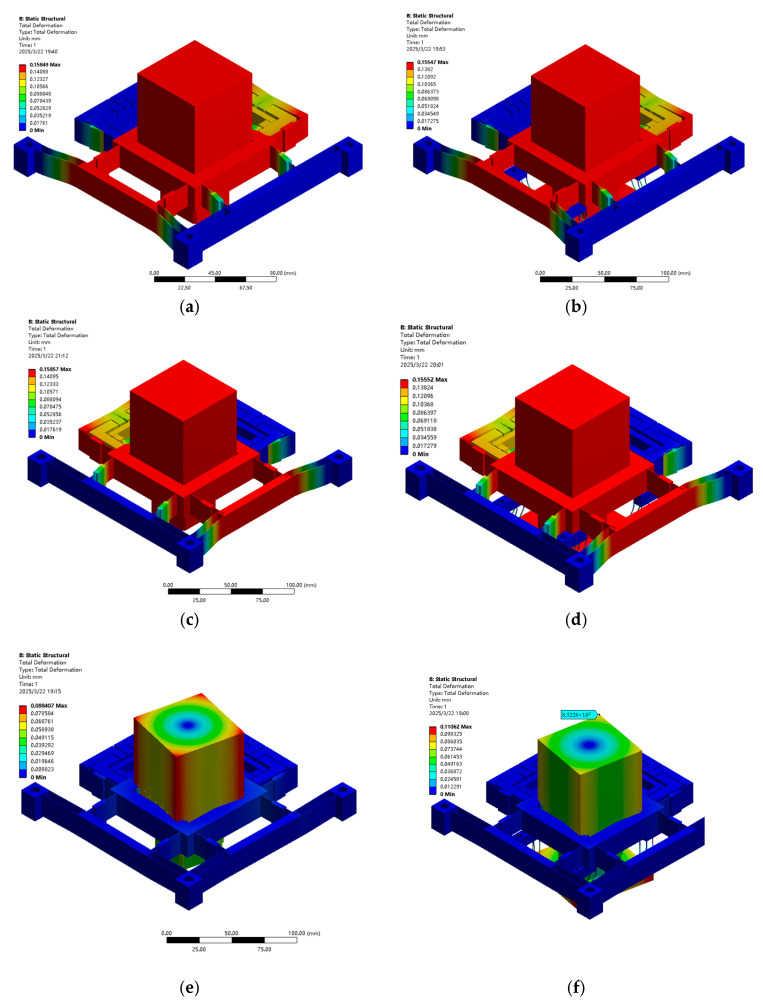
FEA (none) over-constrained mechanism with a 4 KG displacement: (**a**) X-axis without over-constrained mechanism, (**b**) X-axis with over-constrained mechanism (**c**) Y-axis without over-constrained mechanism, (**d**) Y-axis with over-constrained mechanism (**e**) θ_z_-axis without over-constrained mechanism, and (**f**) θ_z_-axis with over-constrained mechanism.

Figure 18 and Figure 19 show how the 20 μm displacement at the XYθ_z_ input terminal, the output stroke and flatness simulation, and the performance improvement of the stage load-bearing line of the over-constrained mechanism, which have been verified. Table 7 presents the improvement in performance (stroke displacement and flatness) post introduction of the over-constrained mechanism along the X, Y, and θ_z_ directions. The FEA results confirm that while the over-constrained mechanism slightly affects the stroke of the X-, Y-, and θ_z_-axes, it substantially enhances flatness performance. This reinforces the effectiveness of the over-constrained design in improving motion accuracy and stability, making it a valuable reference for designing high-precision nano-positioning stages.

### 4.2. Modal Analysis

**Figure 20:**
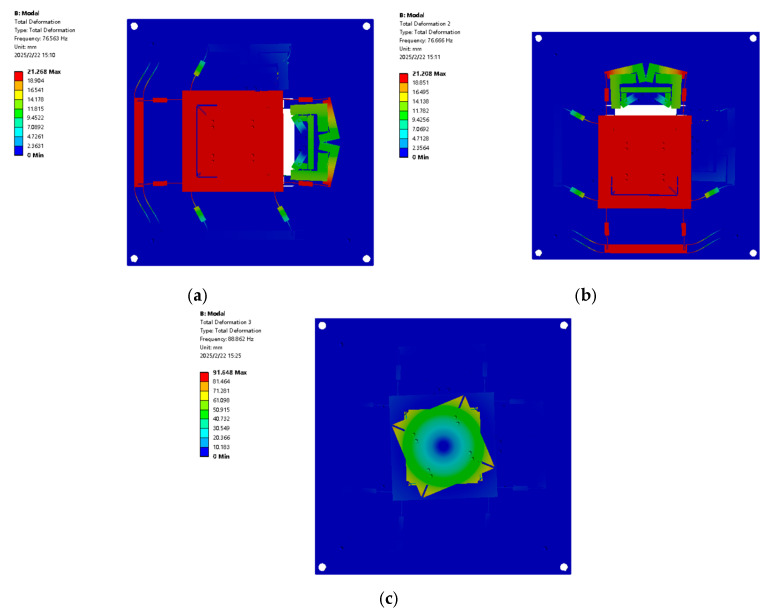
FEA of the dynamic performance XYθ_z_ nano-positioning stage: (**a**) first-order model; (**b**) second-order model; and (**c**) third-order model.

The dynamic performance of the XYθ_z_ nano-positioning stage was studied via FEA using ANSYS for simulating the flexible drive mechanism, and the natural frequency of the corresponding vibration mode was obtained. The material was aluminium alloy, and its properties were the same as that in the static analysis, with a density of 2.81 g/cm^3^. The first three stages of the flexible amplification mechanism were obtained (Figure 20).

Table 8 presents the modal frequencies of the three models. The first-order, second-order, and third-order modal frequencies represent dynamic characteristics related to movement in the X-, Y- and θ_z_-axis directions, respectively.

## 5. Experimental Variation

### 5.1. Experiment Setup

**Figure 21:**
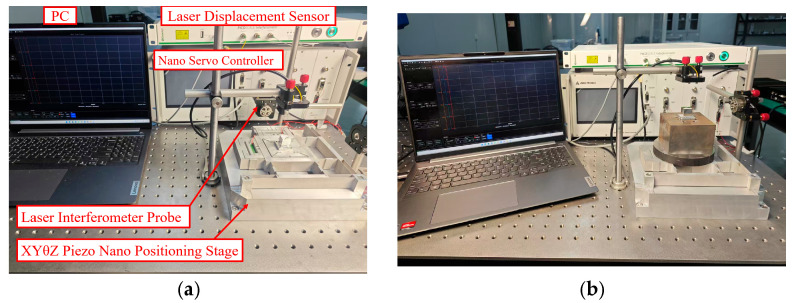
Experimental device: (**a**) stroke experiment and (**b**) load-bearing experiment.

To evaluate the performance of the XYθ_z_ 3-DoF nano-positioning stage and verify the kinematic model, an experimental setup was developed (Figure 21). The stage was made of aluminium alloy AL-7075, processed using electric discharge machining, and placed on an air-floating vibration isolation optical stage.

For actuation, three piezoelectric ceramics (DCS-070736 and DCS-050536, provided by Guangdong Deceratech Co., Ltd., Guangdong, China) were used, driven by a high-bandwidth voltage amplifier (36× magnification). A laser displacement sensor (Smaract GmbH (Oldenburg, Germany)), 1 pm resolution) was used for treal-time measurement of input and output displacements.

### 5.2. Input Output Relationship Test

Under open-loop conditions, a 0–150 V triangular wave signal was applied to the piezoelectric (PZT) actuators along the X-, Y-, and θ_z_-axes, while the output displacement variations were monitored using a laser displacement sensor.

**Figure 22:**
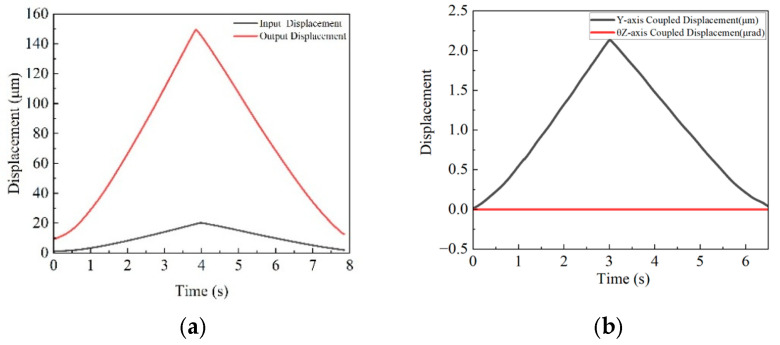
(**a**) X-axis input–output displacement curve; (**b**) X-axis output displacement coupling curve.

Figure 22a shows an X-axis input displacement of 20.1 µm and an output displacement of 152.22 µm, yielding a magnification ratio of 7.57. Figure 22b indicates that during full-stroke X-axis motion, the Y-axis coupling value is 2.1 µm with a 1.37% coupling error, while the θ_z_-axis coupling curve approaches zero, showing minimal impact.

**Figure 23:**
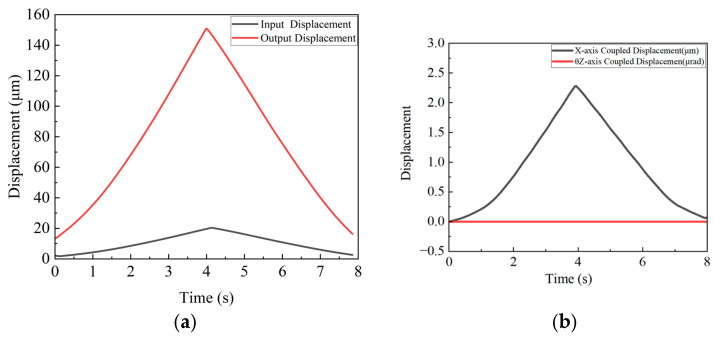
(**a**) Y-axis input–output displacement curve; (**b**) Y-axis output displacement coupling curve.

Figure 23a shows a Y-axis input displacement of 19.8 µm and an output displacement of 151.3 µm, resulting in a magnification ratio of 7.64. Figure 23b demonstrates that during full-stroke Y-axis motion, the X-axis coupling value is 2.2 µm with a coupling error of 1.65%, and the θ_z_-axis coupling curve approaches zero, suggesting negligible influence on the θ_z_-axis.

**Figure 24:**
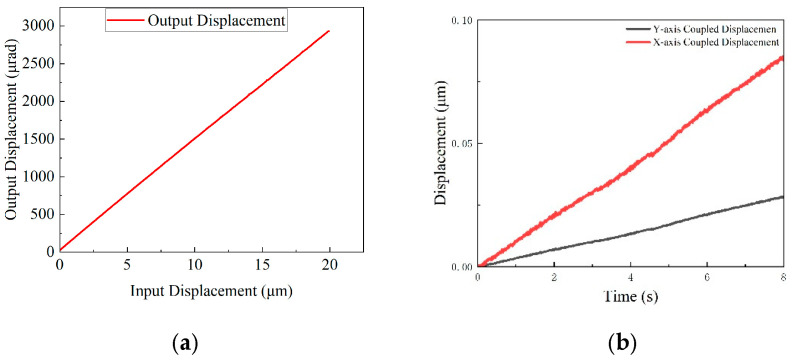
(**a**) θ_z_-axis input–output displacement curve; (**b**) θ_z_-axis output displacement coupling curve.

Figure 24a presents a θ_z_-axis input displacement of 20 µm and an output displacement of 2.885 mrad. Figure 24b shows no periodic changes in the X- and Y-axis coupling curves during full-stroke θ_z_-axis motion, with the observed drift primarily attributed to laser interferometer probe installation vibrations.

Experimental results confirm magnification ratios of 7.57 and 7.64 for the X- and Y- axis, respectively. The FEA comparison shows magnification errors of 1.2% (X) and 2.5% (Y), with a 4.6% max angular deviation in θ_z_, respectively, verifying high consistency between experimental and FEA results and validating model accuracy.

### 5.3. Load-Bearing Test

This experiment aimed to improve the load-bearing capacity and operational stability of the nano-positioning stage through an over-constrained mechanism, with the primary objective of suppressing external force disturbances to ensure consistent and reliable stage performance. The load-bearing experimental system illustrated in Figure 21b was constructed to validate the performance enhancement effect of the over-constrained mechanism on the nano-positioning stage. Comparative experiments were conducted by applying 0–150 V triangular wave signals to the PZT actuators along the X-, Y-, and θ_z_-axes.

Loads of 10 N and 40 N were applied at the centre of the rigid stage, without the over-constrained mechanism. A laser displacement sensor was used to monitor the out-of-plane deformation and displacement responses in real time. Three repeated measurements were conducted for each load, and the average value was recorded.

With the over-constrained mechanism installed, the loading procedure used in Experiment 1 was repeated. The displacement and out-of-plane deformation were recorded using an identical measurement method. The two datasets were compared to evaluate the effect of the over-constrained mechanism on the load-bearing capacity and stability of the nano-positioning stage.

This comparative experimental approach effectively demonstrates the role of the over-constrained mechanism in enhancing mechanical properties of the nano-positioning stage and provides a solid experimental foundation for further structural optimisation.

**Figure 25:**
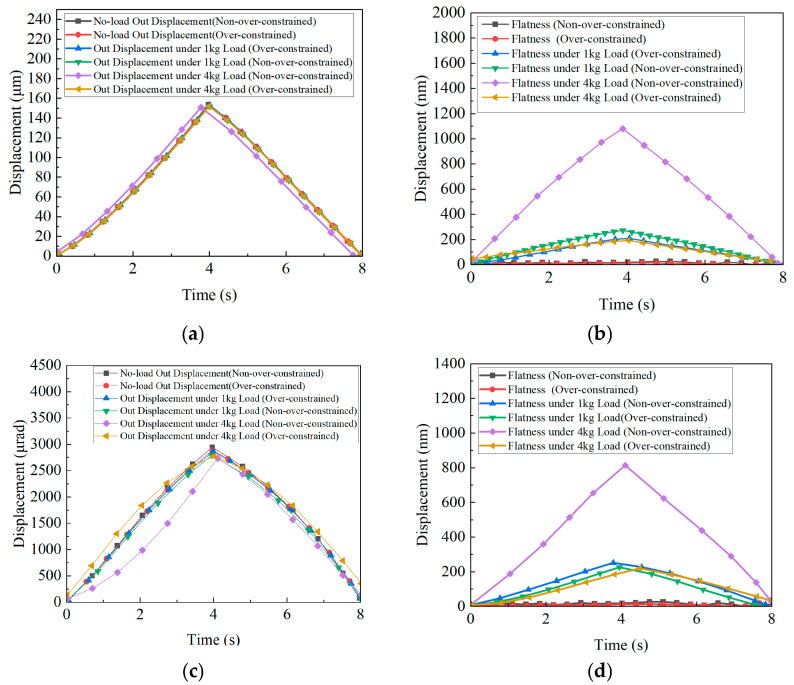
Influence of axial load on displacement and flatness: (**a**) X (Y)-axis displacement change; (**b**) X (Y)-axis flatness change; (**c**) θ_z_-axis displacement change; and (**d**) θ_z_-axis flatness change.

Figure 25a compares the X (Y)-axis displacement curves with and without the over-constrained mechanism under 10 N and 40 N loads. In the no-load state, constrained and unconstrained displacements are 152.22 µm and 153.7 µm, respectively, with nearly identical stroke curves. At 10 N, the unconstrained displacement decreases to 151.96 µm, while the constrained displacement remains at 151.9 µm. At 40 N, the unconstrained displacement drops to 150.73 µm, while the constrained displacement stabilises at 151.4 µm.

Figure 25b illustrates that the flatness curves remain stable under no-load conditions. At 10 N, flatness changes by 262 nm and 208 nm for the unconstrained and constrained mechanisms, respectively. At 40 N, the flatness change in the unconstrained case significantly increases to 1080 nm, while that in the constrained case is limited to 192 nm.

Figure 25c compares θ_z_-axis displacement under 10 N and 40 N loads. In the no-load state, constrained and unconstrained displacements are 2885.211 µrad and 2944.377 µrad, respectively, with similar stroke curves. At 10 N, the unconstrained displacement decreases to 2790.74 µrad, while the constrained displacement changes to 2861.99 µrad. At 40 N, the unconstrained displacement drops to 2732.7 µrad, while the constrained displacement is 2774.93 µrad.

Figure 25d shows that θ_z_-axis flatness remains stable under no-load conditions. At 10 N, flatness changes by 251.447 nm and 225.696 nm for the unconstrained and constrained cases, respectively. At 40 N, the flatness change in the unconstrained case significantly increases to 814.092 nm, while that in the constrained case remains at 219.648 nm.

By leveraging the relationship between force and displacement in piezoelectric ceramics, the input force can be determined. The equation for calculating the input force is $F=K_{PZT}\left(d-y_{i}\right)$, where $K_{PZTxy}=51.5N/\mu m$ and $K_{PZT\theta Z}=25N/\mu m$ represent the equivalent stiffness of the piezoelectric stack, $d_{xy}=38V/150\left(\mu m\right)$ and $d_{\theta Z}=40V/150\left(\mu m\right)$ represent the unloading output displacement of the piezoelectric stack, V represents the input voltage applied to the piezoelectric stack, and $y_{i}$ represents the output displacement of the piezoelectric stack measured with the laser displacement sensor. From Figure 21a, Figure 22a, and Figure 23a, it is evident that with an input voltage of 150 V, the laser interferometer detects an output X/Y/θ_z_ displacement of 20.1 µm, 19.8 µm, and 20.64 µm, respectively. The output force is $F_{x}=921.85\;N$, $F_{y}=937.3\;N$, and $F_{\theta Z}=488.5\;N$. The equivalent load force is input in the FEA, along with the stroke and flatness.

The simulated results of the XYθ_z_ positioning stage are compared with experimental results in Table 9.

Notably, as the load increases from 10 N to 40 N, the decrease in flatness is owing to assembly errors, enabling the over-constrained mechanism to perform better under high loads. At 10 N, differences in stroke and flatness between constrained and unconstrained mechanisms are minimal, as low loads have a limited effect on the axial stiffness of the XYθ_z_ stage.

The results indicate that under these load conditions, displacement and flatness in the motion direction are not significantly affected by axial load changes. The axial bearing stiffness of the stage is independent of motion-direction stiffness. The nano-positioning stage effectively withstands these loads, demonstrating strong load-bearing capacity and stability.

### 5.4. Frequency Characteristics

To determine the first natural frequency of the stage, its frequency response was measured. In the experiment, swept-sine signals were used to excite the PZTs (piezoelectric ceramics) in the X, Y, and θ_z_ directions, respectively, inducing vibrations, and the frequency response signals were captured using a laser sensor.

**Figure 26:**
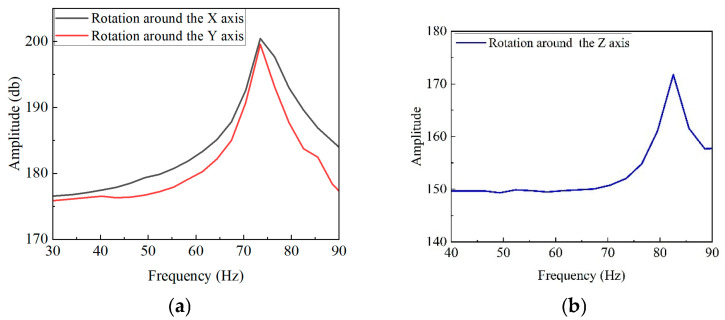
Frequency response experiment results: (**a**) XY-axis frequency response curve and (**b**) θ_z_-axis frequency response curve.

Figure 26 presents the first-order motion resonance frequencies for the X-, Y-, and θz-axes. The X-axis resonance is 72.8 Hz (simulation: 76.563 Hz, error: 4.9%), the Y-axis resonance is 73.49 Hz (simulation: 76.666 Hz, error: 4.1%), and the θ_z_-axis resonance is 82.55 Hz (simulation: 88.86 Hz, error: 7.1%). These errors are attributed to assembly inaccuracies.

To summarise, Table 10 presents a systematic comparison of the proposed XYθ_z_ precision motion stage and its existing counterparts reported in the literature, with a particular focus on key performance indicators including load capacity, stroke, frequency, and displacement coupling error (DCE). The results of the comparison demonstrate that this novel XYθ_z_ stage maintains superior output displacement performance under loaded conditions while exhibiting exceptional loaded motion characteristics.

## 6. Conclusions

This study presented a piezoelectric-driven XYθ_z_ nano-positioning mechanism that integrated a large stroke, high load-bearing capacity, and independent 3-DoF motion. The mechanism comprised parallel translation and rotation modules, enabling precise input–output displacement control through an electromechanical coupling model. Its optimised design enabled wide-range movement in a compact structure. The addition of an over-constrained mechanism enhanced load-bearing capacity, reduced out-of-plane deformation, and ensured flatness stability.

Experimental results confirmed that the prototype (200 mm × 200 mm × 25 mm) achieved X/Y-axis translation strokes of 152.22 µm and 151.3 µm, respectively, and a θ_z_-axis rotation stroke of 2.885 mrad. Under load variations from 10 to 40 N, flatness change remained below 8.5%, and stroke change was within 5%. The output coupling evaluation was under 2%, demonstrating excellent decoupling performance.

These findings indicated that the XYθ_z_ nano-positioning mechanism is well suited for industrial applications that require high load-bearing capacity, large stroke, and high flatness, such as large-area wafer image detection systems. In future research, the XYθ_z_ nano-positioning motion stage should be tested for closed-loop control to further verify its performance.

## Figures and Tables

**Table 1:** Positioning accuracy of each system.

System	Positioning Accuracy
Nanoimprint lithography [5]	6–40 nm
Scanning probe microscopy systems [6]	20 nm
High-precision optical image stabilisation systems [7]	52 nm

**Table 2:** Specific parameters of the guide mechanism.

a (mm)	b (mm)	c (mm)	d (mm)	e (mm)	f (mm)
13	20	0.5	13	38	0.45

**Table 3:** Specific parameters of the over-constrained mechanism.

a (mm)	b (mm)	c (mm)	d (mm)
25	0.5	0.5	7

**Table 4:** Specific parameters of the guide mechanism of part of the θ_z_ stage.

a (mm)	b (mm)	c (mm)
16	20	0.5

**Table 5:** X/θ_z_ amplification mechanism parameter details.

		1	1a	1b	1c	2	3	3a	3b	3c	3d	4	5
X	d (mm)	5	-	-	-	14	-	6.19	7.85	26.7	26.7	23	-
	$\theta_{i}$(°)	0	-	-	-	0	0	-	-	-	-	90	0
θ_z_	d (mm)	-	6.5	3.9	30	3.3	1.5	-	-	-	-	-	-
	$\theta_{i}$(°)	0	-	-	-	0	90	-	-	-		-	

**Table 6:** Comparison of theoretical and FEA results.

Direction	Comparative Performance	FEA	Theoretical	Error
**X**	Amplification	7.665	7.452	2.77%
	Output displacement (μm)	18.443	17.81	3.43%
**Y**	Amplification	7.665	7.452	2.77%
	Output displacement (μm)	18.425	17.82	3.28%
**θ_z_**	Maximum output displacement (μrad)	2890	2755.9	4.64%
	Output displacement (μrad)	447.8333	438.24	2.14%

**Table 7:** Impact of over-constrained mechanism on stage performance in FEA.

	Displacement	Displacement Deviation	Flatness	Flatness Deviation
X	158.49 μm	1.90%	1.5111 μm	84.26%
X (Over-constrained)	155.47 μm		0.2378 μm	
Y	158.57 μm	1.92%	1.5446 μm	87.02%
Y (Over-constrained)	155.52 μm		0.2004 μm	
θ_z_	3031.7667 μrad	4.16%	1.3321 μm	81.94%
θ_z_ (Over-constrained)	2905.6 μrad		0.2405 μm	

**Table 8:** Modal frequencies of X Yθ_z_ nano-positioning stage.

Order	Modal Frequency (Hz)
First-order model	76.563
Second-order model	76.666
Third-order model	88.86

**Table 9:** Comparison of experimental and FEA results for 4 kg load-bearing stage.

Over Constraints	Displacement (X/μm) (θ_z_/μrad)	Error	Flatness (nm)	Error
X (FEA)	157.33	3.7%	200.4	4.19%
X (Experiment)	151.4		192	
θ_z_ (FEA)	2905.6	4.4%	238.8	8.02%
θ_z_ (Experiment)	2774.93		219.648	

**Table 10:** Comparative analysis of the static and dynamic performance of the XYθz precision motion stages reported in the literature.

	Load-Bearing (g)	Storke (μm/μrad)	Frequency (Hz)	DCE
Cai, K. [8]	-	6.9 × 8.5 × 289	522.5 × 628.7 × 629.9	2.91% × 2.52% ×-
Wang, R. [9]	71.72	37.277 × 44.426 × 2152	921 × 921 × 1101	-
Lee, C. [11]	-	122.84 × 108.46 × 685	85.88 × 89.87 × 97.05	-
Wang, G. [13]	1000	32.4 × 25.5 × 40.2	4508.21 × 4590.59 × 4890.59	-
This paper	4000	152.22 × 151.3 × 2885	72.8 × 73.49 × 82.55	1.37% × 1.65% ×-

## Data Availability

The data presented in this study are available upon request from the corresponding author. Specific experimental data have been published in the article.

## Appendix A. Circular Flexible Matrix Element Parameter Expression

$$k=\frac{12+11\mu }{10+10\mu }$$

$$s=\frac{r}{t}$$

$$G=\frac{E}{2\left(1+\mu \right)}$$

$$N_{1}=\frac{\pi \left(2s+1\right)}{\sqrt{4s+1}}-\pi$$

$$N_{2}=\frac{6\pi s^{4}\left(2s+1\right)}{{\left(4s+1\right)}^{5/2}}$$

$$N_{3}=\frac{\pi \left(48s^{5}+8s^{4}+20s^{3}+30s^{2}+10s+1\right)}{4{\left(4s+1\right)}^{5/2}}-\frac{\pi }{4}$$

$$N_{4}=\frac{4s^{4}}{{\left(4s+1\right)}^{2}}$$
